# 13-(*N*,*N*-Di­methyl­amino)­micheliolide 0.08-hydrate

**DOI:** 10.1107/S1600536813030304

**Published:** 2013-11-20

**Authors:** Shobanbabu Bommagani, Narsimha Reddy Penthala, Venumadhav Janganati, Sean Parkin, Peter A. Crooks

**Affiliations:** a Dept. of Pharm. Sciences, College of Pharmacy, University of Arkansas for Medical Sciences, Little Rock, AR 72205, USA; b Dept. of Chemistry, University of Kentucky, Lexington KY 40506, USA

## Abstract

The title compound, C_17_H_27_NO_3_·0.08H_2_O {sytematic name: (3*R*,3a*S*,9*R*,9a*S*,9b*S*)-3-[(di­methyl­amino)­meth­yl]-9-hy­droxy-6,9-dimethyl-3,3a,4,5,7,8,9,9a-octa­hydro­azuleno[4,5-*b*]furan-2(9b*H*)-one 0.08-hydrate}, exhibits intra­molecular O—H⋯O hydrogen bonding to form a ring of graph-set motif *S*(6). As well as this intra­molecular hydrogen bond with the lactone-ring O atom, the hy­droxy H atom forms an O—H⋯O hydrogen bond to the low-occupancy partial water mol­ecule [occupancy = 0.078 (2)]. The water mol­ecule is correlated with disorder of the N(CH_3_)_2_ group [major–minor occupancy factors are 0.922 (2):0.078 (2)]. The dihedral angle between the mean planes of the *trans*-fused seven-membered ring and the lactone ring is 4.42 (9)°.

## Related literature

For the biological activity of 13-*N,N*-di­methyl­amino michel­iolide, see: Rodriguez *et al.* (1976 ▶); Sethi *et al.* (1984 ▶); Acosta & Fixher (1993 ▶); Zhang *et al.* (2012 ▶). For the crystal structure of a similar mol­ecule, see: Acosta *et al.* (1991 ▶). The structure was checked with *PLATON* (Spek, 2009 ▶) and with an *R*-tensor (Parkin, 2000 ▶).
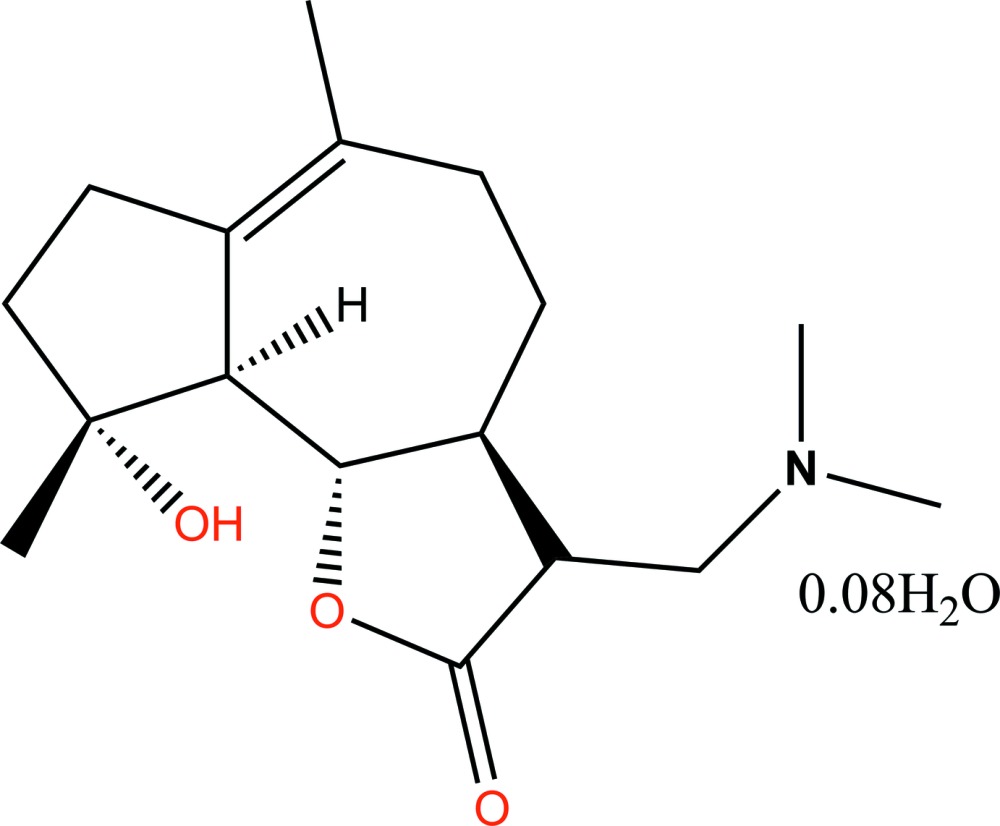


## Experimental

### 

#### Crystal data

C_17_H_27_NO_3_·0.08H_2_O
*M* *_r_* = 295.01
Orthorhombic, 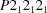

*a* = 9.1329 (2) Å
*b* = 10.5227 (2) Å
*c* = 16.7194 (3) Å
*V* = 1606.78 (5) Å^3^
*Z* = 4
Cu *K*α radiation
μ = 0.66 mm^−1^
*T* = 90 K
0.18 × 0.16 × 0.12 mm

#### Data collection

Bruker X8 Proteum diffractometer
Absorption correction: multi-scan (*SADABS*; Sheldrick, 2008*a* ▶) *T*_min_ = 0.854, *T*_max_ = 0.942
20070 measured reflections
2925 independent reflections
2908 reflections with *I* > 2σ(*I*)
*R*_int_ = 0.032

#### Refinement

*R*[*F* ^2^ > 2σ(*F* ^2^)] = 0.025
*wR*(*F* ^2^) = 0.067
*S* = 1.07
2925 reflections
211 parameters
41 restraints
H-atom parameters constrained
Δρ_max_ = 0.18 e Å^−3^
Δρ_min_ = −0.13 e Å^−3^
Absolute structure: Flack parameter determined using 1227 quotients [(*I* ^+^)−(*I* ^−^)]/[(*I* ^+^)+(*I* ^−^)] (Parsons *et al.*, 2013 ▶)
Absolute structure parameter: −0.01 (3)

### 

Data collection: *APEX2* (Bruker, 2006 ▶); cell refinement: *SAINT* (Bruker, 2006 ▶); data reduction: *SAINT*; program(s) used to solve structure: *SHELXS97* (Sheldrick, 2008*b* ▶); program(s) used to refine structure: *SHELXL2013* (Sheldrick, 2008*b* ▶); molecular graphics: *XP in *SHELXTL** (Sheldrick, 2008*b* ▶); software used to prepare material for publication: *SHELXL2013*.

## Supplementary Material

Crystal structure: contains datablock(s) global, I. DOI: 10.1107/S1600536813030304/sj5365sup1.cif

Structure factors: contains datablock(s) I. DOI: 10.1107/S1600536813030304/sj5365Isup2.hkl

Additional supplementary materials:  crystallographic information; 3D view; checkCIF report

## Figures and Tables

**Table 1:** Hydrogen-bond geometry (Å, °)

*D*—H⋯*A*	*D*—H	H⋯*A*	*D*⋯*A*	*D*—H⋯*A*
O3—H3⋯O2	0.84	2.35	2.9578 (15)	129
O1*W*—H1*W*1⋯O3^i^	0.84	2.16	2.845 (12)	139

Symmetry code: (i) 
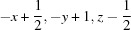
.

**Fig. 1.**
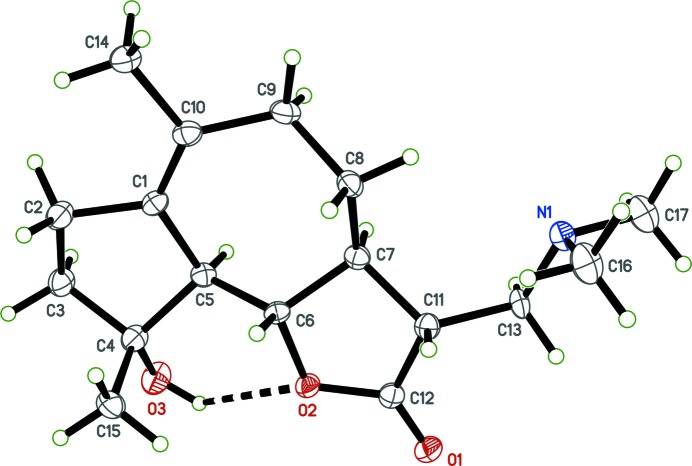
A view of the molecule with displacement ellipsoids drawn at the 50% probability level.

## supplementary crystallographic information

### 1. Comment

Micheliolide (MCL) is a natural product extracted from the plant *Michelia champaca*. MCL belongs to the class of guaianolide sesquiterpene lactones, which are used for the treatment of cancer (Sethi *et al.* 1984). The exocyclic double bond in such sesquiterpenes is believed to be responsible for their biological properties because of its exceptional chemical reactivity with nucleophilic groups (Rodriguez *et al.* 1976). The synthesis of MCL was first reported by Sethi *et al.* (1984) and its crystal structure was described by Acosta *et al.* (1991). Recently, the *N,N*-dimethyl amino analog of micheliolide was reported as a potent anti-leukemic agent (Zhang *et al.* 2012).

The *N,N*-dimethyl amino analog of micheliolide was synthesized as described by Zhang *et al.* (2012). Recrystallization of the compound from hexanes gave colorless needles that were suitable for X-ray analysis. The title molecule, Fig. 1, contains a central seven-membered carbocyclic ring fused to a 5-membered carbocylic ring and a *trans* lactone ring. The two five membered rings are in half-chair conformations, as reported in the literature (Acosta *et al.*, 1991). The X-ray studies revealed that the title compound exhibits an O—H···O intramolecular hydrogen bond between the hydroxyl group and the lactone ring oxygen atom. The structure contains a low occupancy [7.8 (2)% occupancy] partial water molecule and a small amount of correlated disorder of the N(CH_3_)_2_ group. The bridge between the seven-membered ring and the lactone ring is *trans*, giving a dihedral angle between the mean planes of the rings of 4.42 (9)°.

### 2. Experimental

The title compound was prepared by the reported literature procedure (Zhang *et al.*, 2012) and recrystallization from hexanes gave colorless needles suitable for X-ray analysis.

### 3. Refinement

H atoms were found in difference Fourier maps and subsequently placed at idealized positions with constrained distances of 0.98 Å (RCH_3_), 0.99 Å (*R*_2_CH_2_), 1.00 Å (*R*_3_CH) and 0.84 Å (OH). Partial occupancy water H atoms were fixed due to the low occupancy.

*U*_iso_(H) values were set to either 1.2*U*_eq_ or 1.5*U*_eq_ (RCH_3_, OH) of the attached atom. To ensure satisfactory refinement of disordered groups in the structure, a combination of constraints and restraints were employed. The constraints (*SHELXL* commands EADP) were used to fix parameters of superimposed or partially overlapping fragments. Restraints (*SHELXL* commands SAME, SADI, *DFIX* and RIGU) were used to maintain the integrity of ill-defined or disordered groups.

### Crystal data

**Table:**

C_17_H_27_NO_3_·0.08H_2_O	*D*_x_ = 1.220 Mg m^−^^3^
*M**_r_* = 295.01	Cu *K*α radiation, λ = 1.54178 Å
Orthorhombic, *P*2_1_2_1_2_1_	Cell parameters from 9518 reflections
*a* = 9.1329 (2) Å	θ = 5.3–68.3°
*b* = 10.5227 (2) Å	µ = 0.66 mm^−^^1^
*c* = 16.7194 (3) Å	*T* = 90 K
*V* = 1606.78 (5) Å^3^	Block, colourless
*Z* = 4	0.18 × 0.16 × 0.12 mm
*F*(000) = 644	

### Data collection

**Table:**

Bruker X8 Proteum diffractometer	2925 independent reflections
Radiation source: fine-focus rotating anode	2908 reflections with *I* > 2σ(*I*)
Detector resolution: 5.6 pixels mm^-1^	*R*_int_ = 0.032
φ and ω scans	θ_max_ = 68.3°, θ_min_ = 5.3°
Absorption correction: multi-scan (*SADABS*; Sheldrick, 2008*a*)	*h* = −10→8
*T*_min_ = 0.854, *T*_max_ = 0.942	*k* = −12→12
20070 measured reflections	*l* = −16→20

### Refinement

**Table:**

Refinement on *F*^2^	Secondary atom site location: difference Fourier map
Least-squares matrix: full	Hydrogen site location: mixed
*R*[*F*^2^ > 2σ(*F*^2^)] = 0.025	H-atom parameters constrained
*wR*(*F*^2^) = 0.067	*w* = 1/[σ^2^(*F*_o_^2^) + (0.0378*P*)^2^ + 0.270*P*] where *P* = (*F*_o_^2^ + 2*F*_c_^2^)/3
*S* = 1.07	(Δ/σ)_max_ < 0.001
2925 reflections	Δρ_max_ = 0.18 e Å^−^^3^
211 parameters	Δρ_min_ = −0.13 e Å^−^^3^
41 restraints	Absolute structure: Flack parameter determined using 1227 quotients [(*I*^+^)-(*I*^-^)]/[(*I*^+^)+(*I*^-^)] (Parsons *et al.*, 2013)
Primary atom site location: structure-invariant direct methods	Absolute structure parameter: −0.01 (3)

### Special details

**Table:**

Experimental. The crystal was mounted with polyisobutene oil on the tip of a fine glass fibre, fastened in a copper mounting pin with electrical solder. It was placed directly into the cold stream of a liquid nitrogen based cryostat, according to published methods (Hope, 1994; Parkin & Hope, 1998).Diffraction data were collected with the crystal at 90 K, which is standard practice in this laboratory for the majority of flash-cooled crystals.
Geometry. All e.s.d.'s (except the e.s.d. in the dihedral angle between two l.s. planes) are estimated using the full covariance matrix. The cell e.s.d.'s are taken into account individually in the estimation of e.s.d.'s in distances, angles and torsion angles; correlations between e.s.d.'s in cell parameters are only used when they are defined by crystal symmetry. An approximate (isotropic) treatment of cell e.s.d.'s is used for estimating e.s.d.'s involving l.s. planes.
Refinement. Refinement progress was checked using *PLATON* (Spek, 2009) and by an *R*-tensor (Parkin, 2000). The final model was further checked with the IUCr utility *checkCIF*.The partial occupancy water molecule was modelled on the site of a difference map peak of approximately 0.67 e A-3. It must have the same or smaller occupancy as the minor disorder component of disorder in the main molecule, which is a very small amount (less than 8%). Nevertheless, it gave a noticeably better fit, and a much flatter difference map, and so was retained. Hydrogen atoms for this water were placed so as to make reasonable H-bonds to nearby acceptors. Overall, the fit is good and the absolute configuration is well established.

### Fractional atomic coordinates and isotropic or equivalent isotropic displacement parameters (Å^2^)

**Table:**

	*x*	*y*	*z*	*U*_iso_*/*U*_eq_	Occ. (<1)
O1	0.26340 (11)	0.40697 (10)	0.53330 (6)	0.0210 (2)	
O2	0.43064 (11)	0.29124 (9)	0.59801 (6)	0.0165 (2)	
O3	0.42055 (12)	0.12939 (12)	0.74254 (7)	0.0265 (3)	
H3	0.3662	0.1672	0.7098	0.040*	
C1	0.80039 (15)	0.21088 (14)	0.69190 (8)	0.0163 (3)	
C2	0.81758 (16)	0.08807 (15)	0.73919 (9)	0.0214 (3)	
H2A	0.8888	0.0991	0.7833	0.026*	
H2B	0.8513	0.0182	0.7041	0.026*	
C3	0.66481 (17)	0.06029 (15)	0.77202 (9)	0.0213 (3)	
H3A	0.6507	−0.0320	0.7804	0.026*	
H3B	0.6485	0.1051	0.8233	0.026*	
C4	0.56222 (16)	0.11003 (14)	0.70741 (8)	0.0188 (3)	
C5	0.63616 (15)	0.23752 (13)	0.68365 (8)	0.0159 (3)	
H5	0.6086	0.3030	0.7243	0.019*	
C6	0.59247 (14)	0.28641 (14)	0.60217 (8)	0.0147 (3)	
H6	0.6297	0.2268	0.5603	0.018*	
C7	0.63770 (16)	0.42119 (13)	0.58082 (8)	0.0160 (3)	
H7	0.6194	0.4773	0.6280	0.019*	
C8	0.79855 (16)	0.43052 (14)	0.55809 (9)	0.0176 (3)	
H8A	0.8169	0.5145	0.5333	0.021*	
H8B	0.8211	0.3645	0.5177	0.021*	
C9	0.90109 (16)	0.41382 (15)	0.62982 (9)	0.0209 (3)	
H9A	1.0007	0.4391	0.6127	0.025*	
H9B	0.8700	0.4747	0.6717	0.025*	
C10	0.91239 (16)	0.28324 (14)	0.66846 (8)	0.0180 (3)	
C11	0.52508 (16)	0.45244 (14)	0.51589 (8)	0.0169 (3)	
H11	0.5560	0.4102	0.4650	0.020*	
C12	0.39005 (16)	0.38521 (14)	0.54730 (8)	0.0166 (3)	
C14	1.06853 (17)	0.24418 (15)	0.68593 (9)	0.0220 (3)	
H14A	1.1085	0.2982	0.7284	0.033*	
H14B	1.1278	0.2538	0.6375	0.033*	
H14C	1.0704	0.1552	0.7033	0.033*	
C15	0.55145 (18)	0.01621 (14)	0.63836 (9)	0.0233 (3)	
H15A	0.5178	−0.0662	0.6585	0.035*	
H15B	0.6479	0.0062	0.6135	0.035*	
H15C	0.4817	0.0482	0.5986	0.035*	
C13	0.4909 (2)	0.5914 (2)	0.4980 (2)	0.0186 (5)	0.922 (2)
H13A	0.4089	0.5954	0.4593	0.022*	0.922 (2)
H13B	0.4583	0.6334	0.5479	0.022*	0.922 (2)
N1	0.61597 (15)	0.66157 (13)	0.46532 (8)	0.0194 (3)	0.922 (2)
C16	0.6579 (2)	0.6162 (2)	0.38513 (11)	0.0255 (5)	0.922 (2)
H16A	0.6873	0.5268	0.3883	0.038*	0.922 (2)
H16B	0.7399	0.6671	0.3650	0.038*	0.922 (2)
H16C	0.5743	0.6246	0.3488	0.038*	0.922 (2)
C17	0.5765 (2)	0.79590 (17)	0.46031 (11)	0.0294 (4)	0.922 (2)
H17A	0.4966	0.8067	0.4218	0.044*	0.922 (2)
H17B	0.6617	0.8452	0.4428	0.044*	0.922 (2)
H17C	0.5447	0.8259	0.5130	0.044*	0.922 (2)
C13'	0.523 (4)	0.596 (3)	0.494 (3)	0.0186 (5)	0.078 (2)
H13C	0.4480	0.6372	0.5271	0.022*	0.078 (2)
H13D	0.6191	0.6318	0.5094	0.022*	0.078 (2)
N1'	0.4995 (18)	0.6291 (14)	0.4203 (9)	0.0194 (3)	0.078 (2)
C16'	0.656 (3)	0.630 (4)	0.408 (2)	0.0255 (5)	0.078 (2)
H16D	0.6789	0.6777	0.3587	0.038*	0.078 (2)
H16E	0.6917	0.5425	0.4020	0.038*	0.078 (2)
H16F	0.7047	0.6703	0.4533	0.038*	0.078 (2)
C17'	0.439 (3)	0.7572 (17)	0.4127 (14)	0.0294 (4)	0.078 (2)
H17D	0.4467	0.7851	0.3569	0.044*	0.078 (2)
H17E	0.4948	0.8155	0.4470	0.044*	0.078 (2)
H17F	0.3364	0.7569	0.4292	0.044*	0.078 (2)
O1W	0.2432 (13)	0.7614 (11)	0.3700 (7)	0.014 (3)*	0.078 (2)
H1W1	0.1696	0.7606	0.3398	0.022*	0.078 (2)
H2W1	0.2082	0.7455	0.4153	0.022*	0.078 (2)

### Atomic displacement parameters (Å^2^)

**Table:**

	*U*^11^	*U*^22^	*U*^33^	*U*^12^	*U*^13^	*U*^23^
O1	0.0166 (5)	0.0232 (5)	0.0233 (5)	0.0005 (4)	−0.0031 (4)	0.0017 (4)
O2	0.0131 (5)	0.0184 (5)	0.0179 (5)	−0.0009 (4)	−0.0010 (4)	0.0031 (4)
O3	0.0163 (5)	0.0348 (6)	0.0284 (6)	0.0003 (5)	0.0050 (5)	0.0113 (5)
C1	0.0156 (7)	0.0191 (7)	0.0143 (6)	0.0009 (6)	−0.0018 (5)	−0.0010 (6)
C2	0.0178 (7)	0.0245 (7)	0.0219 (7)	0.0011 (6)	−0.0012 (6)	0.0042 (6)
C3	0.0212 (8)	0.0231 (8)	0.0197 (7)	−0.0008 (6)	−0.0002 (6)	0.0056 (6)
C4	0.0155 (7)	0.0234 (7)	0.0176 (7)	−0.0016 (6)	0.0013 (6)	0.0051 (6)
C5	0.0151 (7)	0.0174 (7)	0.0152 (6)	−0.0008 (5)	0.0008 (5)	−0.0002 (5)
C6	0.0109 (6)	0.0171 (6)	0.0162 (6)	−0.0005 (5)	0.0003 (5)	−0.0008 (5)
C7	0.0162 (7)	0.0155 (7)	0.0162 (6)	−0.0001 (5)	0.0005 (6)	−0.0011 (5)
C8	0.0170 (7)	0.0151 (7)	0.0207 (7)	−0.0014 (5)	0.0015 (6)	0.0018 (5)
C9	0.0149 (7)	0.0196 (7)	0.0283 (8)	−0.0038 (6)	−0.0010 (6)	0.0000 (6)
C10	0.0161 (7)	0.0196 (7)	0.0181 (6)	0.0001 (6)	−0.0012 (6)	−0.0036 (6)
C11	0.0173 (7)	0.0169 (7)	0.0166 (6)	0.0004 (5)	0.0005 (5)	−0.0001 (5)
C12	0.0189 (7)	0.0166 (7)	0.0143 (6)	0.0006 (5)	−0.0006 (5)	−0.0019 (5)
C14	0.0172 (7)	0.0224 (7)	0.0266 (7)	−0.0007 (6)	−0.0018 (6)	−0.0001 (6)
C15	0.0261 (8)	0.0198 (7)	0.0240 (7)	−0.0063 (6)	−0.0033 (6)	0.0044 (6)
C13	0.0156 (14)	0.0200 (8)	0.0204 (8)	0.0008 (8)	0.0040 (11)	0.0033 (6)
N1	0.0209 (7)	0.0168 (7)	0.0205 (7)	−0.0012 (5)	0.0002 (5)	0.0032 (5)
C16	0.0328 (8)	0.0234 (10)	0.0202 (13)	0.0006 (7)	0.0104 (8)	0.0018 (10)
C17	0.0363 (10)	0.0179 (8)	0.0340 (9)	−0.0005 (8)	0.0027 (8)	0.0020 (7)
C13'	0.0156 (14)	0.0200 (8)	0.0204 (8)	0.0008 (8)	0.0040 (11)	0.0033 (6)
N1'	0.0209 (7)	0.0168 (7)	0.0205 (7)	−0.0012 (5)	0.0002 (5)	0.0032 (5)
C16'	0.0328 (8)	0.0234 (10)	0.0202 (13)	0.0006 (7)	0.0104 (8)	0.0018 (10)
C17'	0.0363 (10)	0.0179 (8)	0.0340 (9)	−0.0005 (8)	0.0027 (8)	0.0020 (7)

### Geometric parameters (Å, º)

**Table:**

O1—C12	1.2021 (18)	C11—H11	1.0000
O2—C12	1.3542 (17)	C14—H14A	0.9800
O2—C6	1.4804 (15)	C14—H14B	0.9800
O3—C4	1.4355 (18)	C14—H14C	0.9800
O3—H3	0.8400	C15—H15A	0.9800
C1—C10	1.334 (2)	C15—H15B	0.9800
C1—C2	1.523 (2)	C15—H15C	0.9800
C1—C5	1.5321 (18)	C13—N1	1.466 (2)
C2—C3	1.528 (2)	C13—H13A	0.9900
C2—H2A	0.9900	C13—H13B	0.9900
C2—H2B	0.9900	N1—C17	1.461 (2)
C3—C4	1.523 (2)	N1—C16	1.474 (2)
C3—H3A	0.9900	C16—H16A	0.9800
C3—H3B	0.9900	C16—H16B	0.9800
C4—C15	1.522 (2)	C16—H16C	0.9800
C4—C5	1.5536 (19)	C17—H17A	0.9800
C5—C6	1.5098 (18)	C17—H17B	0.9800
C5—H5	1.0000	C17—H17C	0.9800
C6—C7	1.5198 (19)	C13'—N1'	1.29 (5)
C6—H6	1.0000	C13'—C16'	1.92 (5)
C7—C8	1.5206 (19)	C13'—H13C	0.9900
C7—C11	1.531 (2)	C13'—H13D	0.9900
C7—H7	1.0000	N1'—C16'	1.45 (2)
C8—C9	1.532 (2)	N1'—C17'	1.460 (19)
C8—H8A	0.9900	C16'—H16D	0.9800
C8—H8B	0.9900	C16'—H16E	0.9800
C9—C10	1.522 (2)	C16'—H16F	0.9800
C9—H9A	0.9900	C17'—O1W	1.93 (3)
C9—H9B	0.9900	C17'—H17D	0.9800
C10—C14	1.513 (2)	C17'—H17E	0.9800
C11—C12	1.516 (2)	C17'—H17F	0.9800
C11—C13	1.525 (3)	O1W—H1W1	0.8400
C11—C13'	1.55 (2)	O1W—H2W1	0.8400
			
C12—O2—C6	109.14 (11)	C10—C14—H14B	109.5
C4—O3—H3	109.5	H14A—C14—H14B	109.5
C10—C1—C2	123.90 (13)	C10—C14—H14C	109.5
C10—C1—C5	128.30 (14)	H14A—C14—H14C	109.5
C2—C1—C5	107.63 (12)	H14B—C14—H14C	109.5
C1—C2—C3	104.76 (12)	C4—C15—H15A	109.5
C1—C2—H2A	110.8	C4—C15—H15B	109.5
C3—C2—H2A	110.8	H15A—C15—H15B	109.5
C1—C2—H2B	110.8	C4—C15—H15C	109.5
C3—C2—H2B	110.8	H15A—C15—H15C	109.5
H2A—C2—H2B	108.9	H15B—C15—H15C	109.5
C4—C3—C2	103.97 (11)	N1—C13—C11	113.35 (14)
C4—C3—H3A	111.0	N1—C13—H13A	108.9
C2—C3—H3A	111.0	C11—C13—H13A	108.9
C4—C3—H3B	111.0	N1—C13—H13B	108.9
C2—C3—H3B	111.0	C11—C13—H13B	108.9
H3A—C3—H3B	109.0	H13A—C13—H13B	107.7
O3—C4—C15	110.13 (12)	C17—N1—C13	108.44 (15)
O3—C4—C3	108.25 (11)	C17—N1—C16	108.97 (15)
C15—C4—C3	110.78 (13)	C13—N1—C16	112.23 (18)
O3—C4—C5	111.95 (12)	N1—C16—H16A	109.5
C15—C4—C5	113.22 (11)	N1—C16—H16B	109.5
C3—C4—C5	102.16 (12)	H16A—C16—H16B	109.5
C6—C5—C1	113.72 (11)	N1—C16—H16C	109.5
C6—C5—C4	114.20 (12)	H16A—C16—H16C	109.5
C1—C5—C4	104.15 (12)	H16B—C16—H16C	109.5
C6—C5—H5	108.2	N1—C17—H17A	109.5
C1—C5—H5	108.2	N1—C17—H17B	109.5
C4—C5—H5	108.2	H17A—C17—H17B	109.5
O2—C6—C5	108.54 (10)	N1—C17—H17C	109.5
O2—C6—C7	103.19 (11)	H17A—C17—H17C	109.5
C5—C6—C7	117.26 (12)	H17B—C17—H17C	109.5
O2—C6—H6	109.2	N1'—C13'—C11	120 (3)
C5—C6—H6	109.2	N1'—C13'—C16'	49.1 (17)
C7—C6—H6	109.2	C11—C13'—C16'	111 (3)
C6—C7—C8	112.43 (12)	N1'—C13'—H13C	107.4
C6—C7—C11	100.62 (11)	C11—C13'—H13C	107.4
C8—C7—C11	117.25 (12)	C16'—C13'—H13C	141.5
C6—C7—H7	108.7	N1'—C13'—H13D	107.4
C8—C7—H7	108.7	C11—C13'—H13D	107.4
C11—C7—H7	108.7	C16'—C13'—H13D	64.5
C7—C8—C9	112.81 (12)	H13C—C13'—H13D	106.9
C7—C8—H8A	109.0	C13'—N1'—C16'	89 (2)
C9—C8—H8A	109.0	C13'—N1'—C17'	113 (2)
C7—C8—H8B	109.0	C16'—N1'—C17'	111 (2)
C9—C8—H8B	109.0	N1'—C16'—C13'	42.4 (17)
H8A—C8—H8B	107.8	N1'—C16'—H16D	109.5
C10—C9—C8	118.52 (12)	C13'—C16'—H16D	148.7
C10—C9—H9A	107.7	N1'—C16'—H16E	109.5
C8—C9—H9A	107.7	C13'—C16'—H16E	95.9
C10—C9—H9B	107.7	H16D—C16'—H16E	109.5
C8—C9—H9B	107.7	N1'—C16'—H16F	109.5
H9A—C9—H9B	107.1	C13'—C16'—H16F	77.4
C1—C10—C14	120.73 (13)	H16D—C16'—H16F	109.5
C1—C10—C9	126.02 (13)	H16E—C16'—H16F	109.5
C14—C10—C9	113.03 (12)	N1'—C17'—O1W	113.7 (15)
C12—C11—C13	110.41 (12)	N1'—C17'—H17D	109.5
C12—C11—C7	101.57 (11)	O1W—C17'—H17D	72.8
C13—C11—C7	118.86 (16)	N1'—C17'—H17E	109.5
C12—C11—C13'	121.9 (15)	O1W—C17'—H17E	132.9
C7—C11—C13'	112.7 (18)	H17D—C17'—H17E	109.5
C12—C11—H11	108.5	N1'—C17'—H17F	109.5
C13—C11—H11	108.5	O1W—C17'—H17F	38.1
C7—C11—H11	108.5	H17D—C17'—H17F	109.5
O1—C12—O2	121.62 (13)	H17E—C17'—H17F	109.5
O1—C12—C11	128.80 (13)	C17'—O1W—H1W1	164.8
O2—C12—C11	109.58 (12)	C17'—O1W—H2W1	90.8
C10—C14—H14A	109.5	H1W1—O1W—H2W1	103.6
			
C10—C1—C2—C3	164.39 (14)	C8—C9—C10—C1	−50.3 (2)
C5—C1—C2—C3	−11.20 (15)	C8—C9—C10—C14	135.02 (14)
C1—C2—C3—C4	33.25 (15)	C6—C7—C11—C12	36.38 (13)
C2—C3—C4—O3	−160.27 (12)	C8—C7—C11—C12	158.62 (12)
C2—C3—C4—C15	78.88 (15)	C6—C7—C11—C13	157.67 (14)
C2—C3—C4—C5	−42.00 (14)	C8—C7—C11—C13	−80.10 (18)
C10—C1—C5—C6	45.2 (2)	C6—C7—C11—C13'	168.5 (17)
C2—C1—C5—C6	−139.44 (12)	C8—C7—C11—C13'	−69.3 (17)
C10—C1—C5—C4	170.15 (14)	C6—O2—C12—O1	179.20 (13)
C2—C1—C5—C4	−14.51 (15)	C6—O2—C12—C11	−1.67 (15)
O3—C4—C5—C6	−85.21 (15)	C13—C11—C12—O1	29.4 (2)
C15—C4—C5—C6	40.01 (17)	C7—C11—C12—O1	156.38 (15)
C3—C4—C5—C6	159.18 (11)	C13'—C11—C12—O1	30 (2)
O3—C4—C5—C1	150.16 (11)	C13—C11—C12—O2	−149.67 (17)
C15—C4—C5—C1	−84.62 (14)	C7—C11—C12—O2	−22.67 (14)
C3—C4—C5—C1	34.56 (14)	C13'—C11—C12—O2	−149 (2)
C12—O2—C6—C5	150.78 (11)	C12—C11—C13—N1	−178.49 (19)
C12—O2—C6—C7	25.69 (13)	C7—C11—C13—N1	64.8 (3)
C1—C5—C6—O2	172.18 (11)	C13'—C11—C13—N1	5 (10)
C4—C5—C6—O2	52.82 (15)	C11—C13—N1—C17	−172.80 (19)
C1—C5—C6—C7	−71.48 (16)	C11—C13—N1—C16	66.8 (3)
C4—C5—C6—C7	169.16 (12)	C12—C11—C13'—N1'	−94 (3)
O2—C6—C7—C8	−163.62 (11)	C13—C11—C13'—N1'	−91 (11)
C5—C6—C7—C8	77.15 (15)	C7—C11—C13'—N1'	144 (3)
O2—C6—C7—C11	−38.06 (13)	C12—C11—C13'—C16'	−148.3 (17)
C5—C6—C7—C11	−157.29 (12)	C13—C11—C13'—C16'	−145 (12)
C6—C7—C8—C9	−71.33 (15)	C7—C11—C13'—C16'	91 (3)
C11—C7—C8—C9	172.75 (12)	C11—C13'—N1'—C16'	−93 (3)
C7—C8—C9—C10	69.48 (17)	C11—C13'—N1'—C17'	155 (2)
C2—C1—C10—C14	1.9 (2)	C16'—C13'—N1'—C17'	−112 (2)
C5—C1—C10—C14	176.57 (13)	C17'—N1'—C16'—C13'	114 (2)
C2—C1—C10—C9	−172.40 (13)	C13'—N1'—C17'—O1W	−115 (2)
C5—C1—C10—C9	2.2 (2)	C16'—N1'—C17'—O1W	147.4 (19)

### Hydrogen-bond geometry (Å, º)

**Table:**

*D*—H···*A*	*D*—H	H···*A*	*D*···*A*	*D*—H···*A*
O3—H3···O2	0.84	2.35	2.9578 (15)	129
O1*W*—H1*W*1···O3^i^	0.84	2.16	2.845 (12)	139

Symmetry code: (i) −*x*+1/2, −*y*+1, *z*−1/2.

## References

[bb1] Acosta, J. & Fixher, N. (1993). *J. Nat. Prod.* **56**, 90–98.

[bb2] Acosta, J. C., Fronczek, F. R. & Fischer, N. H. (1991). *Acta Cryst.* C**47**, 2702–2704.

[bb3] Bruker (2006). *APEX2* and *SAINT* Bruker AXS Inc., Madison, Wisconsin, USA.

[bb4] Parkin, S. (2000). *Acta Cryst.* A**56**, 157–162. 10.1107/s010876739901497x 10772457

[bb5] Parsons, S., Flack, H. D. & Wagner, T. (2013). *Acta Cryst.* B**69**, 249–259. 10.1107/S2052519213010014 PMC3661305 23719469

[bb6] Rodriguez, E., Towers, G. H. N. & Mitchell, J. C. (1976). *Phytochemistry*, **15**, 1573–1580.

[bb7] Sethi, V. K., Thappat, R. K., Dhar, K. L. & Atal, C. K. (1984). *Planta Med.* **50**, 364. 10.1055/s-2007-969739 6505094

[bb8] Sheldrick, G. M. (2008*a*). *Acta Cryst.* **A**64, 112–122.

[bb9] Sheldrick, G. M. (2008*b*). *SADABS* University of Göttingen, Germany.

[bb10] Spek, A. L. (2009). *Acta Cryst.* **D**65, 148–155.

[bb11] Zhang, Q., Lu, Y., Ding, Y., Zhai, J., Ji, Q., Ma, W., Yang, M., Fan, H., Long, J., Tong, Z., Shi, Y., Jia, Y., Han, B., Zhang, W., Qiu, C., Ma, X., Li, Q., Shi, Q., Zhang, H., Li, D., Zhang, J., Lin, J., Li, L. Y., Gao, Y. & Chen, Y. (2012). *J. Med. Chem.* **55**, 8757–8769. 10.1021/jm301064b 22985027

